# Diagnostic performance of contrast enhanced CT and ^18^F-FDG PET/CT in suspicious recurrence of biliary tract cancer after curative resection

**DOI:** 10.1186/1471-2407-11-188

**Published:** 2011-05-21

**Authors:** Yun-Gyoo Lee, Sae-Won Han, Do-Youn Oh, Eui Kyu Chie, Jin-Young Jang, Seock-Ah Im, Tae-You Kim, Sun-Whe Kim, Sung Whan Ha, Yung-Jue Bang

**Affiliations:** 1 Department of Internal Medicine, Seoul National University Hospital, Seoul, Republic of Korea; 2 Department of Radiation Oncology, Seoul National University Hospital, Seoul, Republic of Korea; 3 Department of Surgery, Seoul National University Hospital, Seoul, Republic of Korea; 4 Cancer Research Institute, Seoul National University College of Medicine, Seoul, Republic of Korea; 5 Department of Molecular Medicine & Biopharmaceutical Sciences, Graduate School of Convergence Science and Technology, Seoul National University, Seoul, Republic of Korea

**Keywords:** ^18^F-FDG PET/CT, biliary tract cancer, surveillance, recurrence

## Abstract

**Background:**

Because of the late clinical presentation of biliary tract cancer (BTC), only 10% of patients are eligible for curative surgery. Even among those patients who have undergone curative surgery, most patients develop recurrent cancer. This study is to determine the clinical role of ^18^F-FDG PET/CT during post-operative surveillance of suspected recurrent BTC based on symptoms, laboratory findings and contrast-enhanced CT (ceCT) findings.

**Methods:**

We consecutively enrolled 50 patients with BTC who underwent curative surgery. An ^18^F-FDG PET/CT was obtained for assessment of recurrence based on clinical suspicion during post-operative surveillance. The final confirmation of recurrence was determined pathologically or clinically. When a pathologic confirmation was impossible or inconclusive, a clinical confirmation was used by radiologic correlation with subsequent follow-up ceCT at a minimum of 3-month intervals. Diagnostic efficacy was evaluated by comparing the results of ceCT and ^18^F-FDG PET/CT with the final diagnosis.

**Results:**

Among the 50 patients, 34(68%) were confirmed to have a recurrence. PET/CT showed higher sensitivity (88% *vs*. 76%, *p *= 0.16) and accuracy (82% *vs*. 66%, *p *= 0.11) for recurrence compared to ceCT, even though the difference was not significant. The positive (86% *vs*. 74%, *p *= 0.72) and negative predictive values for recurrence (73% *vs*. 47%, *p *= 0.55) were not significantly different between PET/CT and ceCT. However, an additional PET/CT on ceCT significantly improved the sensitivity than did a ceCT alone (94% [32/34] for PET/CT on ceCT *vs*. 76% [26/34] for ceCT alone, *p *= 0.03) without increasing the specificity, positive predictive value, and negative predictive value.

**Conclusions:**

^18^F-FDG PET/CT alone is not more sensitive or specific than ceCT in the detection of recurrent BTC after curative surgery. These results do not reach statistical significance, probably due to the low number of patients. However, an additional ^18^F-FDG PET/CT on ceCT significantly improves the sensitivity of detecting recurrences.

## Background

Biliary tract cancers (BTCs) are rare, highly lethal carcinomas that develop from the epithelium of the gallbladder and bile duct. Because of the late clinical presentation of BTCs, only 10% of patients are eligible for curative surgery [1,2]. Even among those patients who have undergone curative surgery, a majority of the patients develop recurrent cancer, supporting the notoriously poor prognosis of BTCs. Nevertheless, the prompt diagnosis of recurrent BTC could allow for prompt treatment and reduce the number of unnecessary interventions [2]. Therefore, the surveillance of BTC recurrence is important.

According to the practice guidelines for detecting BTC recurrences, a physical examination with routine laboratory tests, every 3-4 months for the first 3 years after surgery and then every 6 months until the 5th post-operative year, is recommended [3]. Additionally, because of the high risk of recurrence, it is recommended that surveillance includes radiologic evaluation with an abdominal CT scan every 6 months for 2-3 years [3,4]. The role of tumor marker (CA19-9) as surveillance is not clear, but constantly rising levels often precede radiologic evidence of recurrence by a number of months [3].

During surveillance, the definitive diagnosis of suspicious lesions on contrast-enhanced CT (ceCT) scans as recurrent cancer can only be made by pathologic confirmation. However, a valid biopsy is not always possible as the lesion of interest is often small in size and located deep adjacent to large vessels or vital structures. There are also cases in which no definite recurrent lesions are detected on ceCT following measurement of elevated tumor markers, such as CA19-9 or CEA, with or without suspicious symptoms. In these cases, recurrence status must only be determined clinically by considering the clinical context, including symptoms, laboratory findings, and CT findings.

Recently, ^18^F-fluorodeoxyglucose positron emission tomography integrated with computed tomography (^18^F-FDG PET/CT) has been introduced and considered to be a valuable imaging tool by combining anatomic and metabolic imaging information in cancer [5]. In particular, the usefulness of ^18^F-FDG PET/CT is reported in several studies for staging of breast, colorectal, lung, and head and neck cancers, cholangiocarcinomas, lymphomas, and for detecting recurrences of breast, colorectal, lung and cervical cancers [6-12]. However, a study concerning the role of ^18^F-FDG PET/CT during surveillance of BTCs is scarce [13]. Therefore, we conducted this study to evaluate and compare the diagnostic validity of ceCT and ^18^F-FDG PET/CT in the assessment of BTC recurrence after curative surgery. In addition, we searched for correlation between maximal standardized uptake value (SUV_max_) in ^18^F-FDG PET/CT and tumor markers.

## Methods

### Patients

Between October 2003 and June 2008 we consecutively enrolled 50 patients with BTCs who underwent curative resection at Seoul National University Hospital and obtained a ^18^F-FDG PET/CT for assessment of recurrence due based on clinical suspicion. All data were collected and analyzed retrospectively.

Patients were classified as clinically suspicious for recurrent BTC and underwent ^18^F-FDG PET/CT scans when there is at least one of the following criteria: 1) suspicious CT findings, 2) elevated tumor markers, such as CA19-9 or CEA, 3) abnormal liver function tests, and/or 4) suspicious clinical symptoms.

### Contrast enhanced CT

All CT images were obtained using a multidetector-row computed tomography (MDCT) scanner (Mx 8000, Philips Healthcare; or LightSpeed Ultra, GE Healthcare; Sensation 16, Simens Healthcare). Each patient received 90 mL of nonionic contrast material that was administered at a rate of 3.0-4.0 mL/s using a mechanical injector. For the MDCT examinations, 3- to 5-mm slice thickness, and 3- to 5-mm reconstruction interval were used. CT images were obtained during the arterial and portal venous phases.

### ^18^F-FDG PET/CT

All scans were performed with one of two PET/CT systems (Philips Gemini Dual; Best, The Netherlands [from October 2003 to the end of the study] or SIMENS Biograph TruePoint; Germany [from November 2007 to the end of the study]). After fasting for 8 hours, 5.18 MBq/kg (0.14 mCi/kg) of FDG was injected. Then, all of the subjects rested quietly for 1 hour. A whole-body ^18^F-FDG PET scan was performed from the skull base to the mid-thigh. The CT scan without contrast was performed immediately prior to the PET scan with a multi-detector 2-slice spiral CT scanner. For the whole-body emission scan, 9-bed positions were examined at 3 min per step. CT and PET images were reconstructed onto a 512 × 512 matrix and a 128 × 128 matrix, respectively, and integrated after attenuation correction.

### Data analysis and statistical methods

Two other board-certified radiologists, who were blinded to the diagnosis, independently interpreted ceCT images for the recurrence. All lesions on the ceCT were interpreted as recurrent, equivocal, or benign. The appearance of new malignant lesions in locoregional area or distant site denotes disease recurrence. For the confirmation of recurrence by ceCT, the RECIST criteria were used [14]. When a postoperative image finding was uneventful: no locoregional recurrence and no distant metastasis, we can define a benign finding. If we cannot differentiate recurrence from benign, the lesion of interest is an equivocal finding. On statistical analysis, equivocal lesions were regarded as benign. Abnormal ^18^F-FDG PET/CT lesions were assessed as benign or malignant with respect to their location, patterns of uptake, and SUV_max_. Recurrence in the liver remnant or operative site was categorized as locoregional recurrence. In addition, recurrences in other areas were categorized as metastases.

When evaluating findings of ^18^F-FDG PET/CT on ceCT as recurrences, at least one image finding of recurrence was necessary. However, benign findings on both ^18^F-FDG PET/CT and ceCT were necessary to interpret the lesions of interest as benign.

Either histopathologic or clinical confirmation after ^18^F-FDG PET/CT was considered the standard of reference. When a pathologic confirmation was possible, it was considered the standard of reference. However, when pathologic confirmation was impossible or inconclusive, we resorted to a clinical confirmation via radiologic correlation with subsequent ceCT with a minimum 3-month follow-up. Thus, no significant interval change for at least 3-months of follow-up by ceCT was required to confirm the lesion of interest as clinically benign. If there is a significant increase in size of lesion of interest, we can confirm the lesion is malignant.

The diagnostic validity of ceCT, ^18^F-FDG PET/CT, and the combination was evaluated by comparison with the standard of reference. Overall and site-specific sensitivity, specificity, positive predictive value, and negative predictive value were calculated. McNemar's test and Fisher's exact test were used to evaluate the efficacy of ceCT, ^18^F-FDG PET/CT, and the combination. Receiver operating characteristics (ROC) analysis was performed for the detection of recurrent lesions of ceCT and ^18^F-FDG PET/CT. All *p *values were two-sided in tests and *p *values less than or equal to 0.05 were considered to be statistically significant. All analyses were performed using Stata 9.0 software (Stata Corp, College Station, TX, USA).

### Ethics

The study protocol was reviewed and approved by the Institutional Review Board of Seoul National University Hospital (IRB No: 1001-001-304). All studies were carried out according to the Declaration of Helsinki guidelines for biomedical research.

## Results

### Patients

Fifty patients were enrolled. The patient characteristics are shown in Table 1. Males comprised 74% of the study patients (n = 37). The locations of tumors were as follows: intrahepatic, 24%; common bile duct, 40%; ampullar of Vater, 28% and gallbladder, 8%. According to the initial pathologic staging, 36% of the patients were node-positive (≥ stage IIB in AJCC 6^th ^classification) [15]. The 15 of 22 patients of observation group (68.2%) were recurred. Interestingly, 19 of 28 patients of adjuvant treatment group (67.9%) were recurred.

**Table 1:** Clinicopathologic Characteristics of Patients

Characteristics		No. of patients (N = 50)
Age	Mean (range)	60 years (33-77)
Gender	Male: Female	37 (74%): 13 (26%)
Tumor type	Intrahepatic cholangiocarcinoma	12 (24%)
	CBD cancer	20 (40%)
	Ampulla of Vater cancer	14 (28%)
	Gallbladder cancer	4 (8%)
Pathologic stage		
Intrahepatic	I	9 (18%)
	IIIC	3 (6%)
Extrahepatic	IA	3 (6%)
	IB	10 (20%)
	IIA	10 (20%)
	IIB	14 (28%)
	III	1 (2%)
Tumor differentiation	Well differentiated	8 (16%)
	Moderate differentiated	34 (68%)
	Poorly differentiated	4 (8%)
	Not classified	4 (8%)
Post-operative treatment	Observation	22 (44%)
	Adjuvant chemotherapy	3 (6%)
	Adjuvant chemoradiation	25 (50%)
Interval between post-operative treatment and suspicion of recurrence	Median (range)	10.7 months (0.5-97.3)
Interval between ceCT and PET/CT	Median (range)	17 days (1-70)

Median time interval between ceCT and ^18^F-FDG PET/CT was 17 days (range 1-70) and 17 patients had the longer interval than 3 weeks. In all 50 patients, median overall survival was 52.5 months (95% CI: 41.1, -). The median follow-up time was 49.1 months (range 5.1-86, standard error 11.5).

Before ^18^F-FDG PET/CT was undertaken, there had been suspicious CT lesions for recurrence in 30 patients. Among 17 patients, there was at least 1 other suspicious finding (elevated tumor marker, symptoms, and abnormal liver function) in addition to suspicious CT findings (Table 2).

**Table 2:** Clinical Suspicion before ^18^F-FDG PET/CT

Suspicious CT findings	Elevated tumor markers	Concerning clinical symptoms*	Abnormal liver function tests†	No. of patients (N = 50)
**+**	-	-	-	30 (60%)
**+**	**+**	-	-	10 (20%)
**+**	-	**+**	-	3 (6%)
**+**	-	-	**+**	3 (6%)
**+**	**+**	**+**	**+**	1 (2%)
-	**+**	-	-	2 (4%)
-	**+**	**+**	-	1 (2%)

* Significant abdominal pain

**† **Reflecting cholestasis

### Detection of Recurrence by ^18^F-FDG PET/CT and ceCT

The median interval between ceCT and ^18^F-FDG PET/CT was 17 days (range, 1-70 days). The recurrence status was determined in 6 patients based on pathologic assessment and in 44 patients based on clinical findings. Among the 50 patients, 34 had confirmed recurrences. Only 6 patients were eligible for pathologic confirmation (5 by biopsy, 1 by surgery): 4 lesions were recurred malignancies and 1 lesion was benign. However, a biopsy of the other 1 lesion was inconclusive because of limited gain of specimen. In 39 recurrent lesions (multiples included), 16 were recurrent near the locoregional area and 16 were recurrent in the lymph nodes (Table 3).

**Table 3:** Detection of recurrence

		No. of patients (N = 50)
Recurrence	Yes	34 (68%)
	No	16 (32%)
Pathologic confirmation		6 (12%)
Clinical confirmation		44 (88%)

		No. of lesions (n = 39)

Recurrence site (multiple counted)	Locoregional recurrence (Liver or anastomosis site)	16 (41%)
	Distant metastasis	23 (60%)
	Lymph node	16 (42%)
	Peritoneum	3 (8%)
	Other (Lung, bone)	4 (10%)

Overall and site-specific sensitivity, specificity, positive predictive value, and negative predictive value according to each image modality are presented in Tables 4 and 5. PET/CT showed higher sensitivity and accuracy compared to ceCT, but the results did not reach statistical significance. While an additional PET/CT on ceCT significantly improved the overall sensitivity (*p *= 0.03), the overall diagnostic efficacy between ceCT alone and PET/CT alone, or ceCT alone and PET/CT on ceCT, was not significantly different. The lower specificity in combination of ceCT and PET/CT than ceCT or PET/CT alone can be explained that benign findings from both ceCT and PET/CT were necessary to interpret the lesions of interest as benign: among 7 patients identified as benign in ceCT and 11 patients identified as benign in PET/CT, only 6 patients with benign findings from both ceCT and PET/CT (Table 4). In addition, the site-specific diagnostic efficacy did not show any significant difference between each diagnostic modality (Table 5).

**Table 4:** Diagnosis of tumor recurrence by ceCT and ^18^F-FDG PET/CT

	ceCT	PET/CT	Combination of ceCT and PET/CT	*p*-value	
				
				ceCT *vs *PET/CT	ceCT *vs *Combination
Sensitivity*	26/34 (76%)	30/34 (88%)	32/34 (94%)	0.16	**0.03**
Specificity*	7/16 (44%)	11/16 (69%)	6/16 (38%)	0.10	1.00
PPV	26/35 (74%)	30/35 (86%)	32/42 (76%)	0.72	1.00
NPV	7/15 (47%)	11/15 (73%)	6/8 (75%)	0.55	1.00
Accuracy	33/50 (66%)	41/50 (82%)	38/50 (76%)	0.11	0.38

*p-value was calculated by McNemar's test

PPV = positive predictive value; NPV = negative predictive value

**Table 5:** Site-specific efficacy of ceCT and ^18^F-FDG PET/CT

Site		ceCT	PET/CT	Combination of ceCT and PET/CT	*p*-value	
					
					ceCT *vs *PET/CT	ceCT *vs *Combination
	Sensitivity*	88% (14/16)	88% (14/16)	100% (16/16)	0.10	0.5
Locoregional area (n = 16)	Specificity*	86% (37/43)	93% (40/43)	84% (36/43)	0.38	1.0
	PPV	70% (14/20)	82% (14/17)	70% (16/23)	0.46	1.0
	NPV	95% (37/39)	93% (40/42)	100% (36/36)	1.0	0.49
	Sensitivity*	63% (10/16)	94% (15/16)	94% (15/16)	0.08	0.08
Lymph node (n = 16)	Specificity*	93% (40/43)	95% (41/43)	91% (39/43)	0.91	0.74
	PPV	77% (10/13)	88% (15/17)	79% (15/19)	0.63	0.61
	NPV	87% (40/46)	98% (41/43)	98% (39/40)	0.11	0.12

* *p*-value was calculated by McNemar's test

PPV = positive predictive value; NPV = negative predictive value

**Figure 1:**
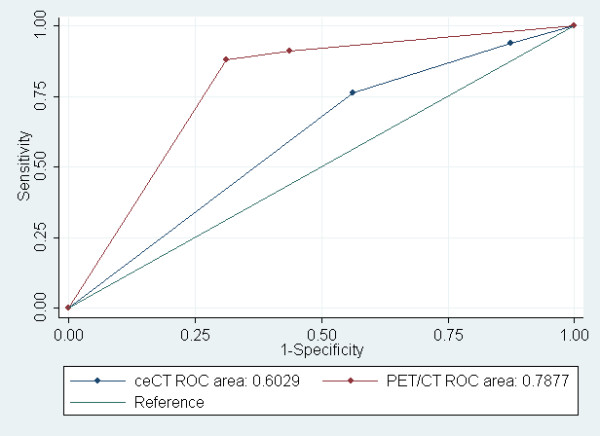
**Receiver Operating Characteristic Curve of ceCT and PET/CT for Detection of Recurrence**.

The ROC analysis indicated that a PET/CT had the higher overall accuracy for the detection of recurrence than ceCT (AUC 0.788 in PET/CT *vs*. AUC 0.603 in ceCT; Figure 1).

### Tumor marker and^18^F-FDG uptake

When there was an indication for ^18^F-FDG PET/CT, 14 patients had elevated CA19-9 levels compared to post-operative levels (Table 2). Among 14 patients, 11 were confirmed to have recurrences. Although the CA19-9 level was elevated, the difference in mean SUV_max _in the lesion of interest was not statistically significant (*p *= 0.42).

The mean SUV_max _of recurrent sites was 4.30 (95% CI, 3.04-5.36), which was higher than 2.52 (95%CI, 1.45-3.58) of the non-recurrent sites and of borderline significance (*p *= 0.06). However, the mean SUV_max _by each recurrent site was similar (4.53 in locoregional areas, 4.4 in lymph node areas). Among 34 patients with recurrent disease, we calculated the median value of SUV_max _(3.35) and compared overall survival between patients with SUV_max _of 3.35 or less and greater than 3.35. However, the survival probability for patients with SUV_max _of 3.35 or less was not significantly higher than patients with SUV_max _greater than 3.35 (*p *= 0.40).

### Clinical decisions based on imaging findings

**Figure 2:**
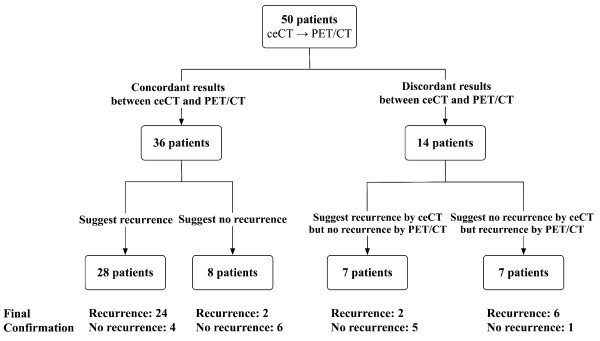
**Flow Diagram Showing Identification of Recurrence based on Imaging Finding**.

Figure 2 presents a flow diagram showing the identification of recurrences based on imaging findings. Of the 50 study patients, 36 lesions had concordant results between ceCT and ^18^F-FDG PET/CT (total agreement, 72%; kappa statistics = 0.33). For 14 lesions with discordant results, ^18^F-FDG PET/CT was 79% identical to the final recurrence status (11 of 14). In contrast, ceCT was 21% identical to the final recurrence status (3 of 14).

## Discussion

The objective of the current study was to determine the clinical role of ^18^F-FDG PET/CT in the assessment of BTC recurrences when clinicians suspected recurrence during surveillance based on symptoms, laboratory findings (including tumor markers), and CT findings. Our data suggest that ^18^F-FDG PET/CT alone is not more sensitive or specific than ceCT in the detection of recurrent BTCs. However, these results do not reach statistical significance, probably due to the low number of patients. On the other hand, an additional ^18^F-FDG PET/CT on ceCT significantly improves the sensitivity in detecting recurrences.

Previous studies have evaluated the utility of ^18^F-FDG PET or PET/CT in BTCs [11,13,16-18]. The study by Kim *et al *enrolled 123 patients with suspected and potentially operable cholangiocarcinomas, and demonstrated that PET/CT showed no advantage over ceCT in the diagnosis of primary tumors, but was more valuable in the diagnosis of regional lymph node and distant metastases [11]. Petrowsky *et al *reported that PET/CT and ceCT provided comparable accuracy for primary cholangiocarcinoma, but PET/CT was particularly valuable in detecting unsuspected distant metastases which were not diagnosed by standard imaging in the staging of patients with gallbladder cancer and cholangiocarcinomas (12/12 in PET/CT *vs*. 3/12 in ceCT; *p *< 0.001) [17]. In these studies, ^18^F-FDG PET or PET/CT did not have a statistically significant advantage over ceCT in the diagnosis of primary tumors, but was valuable in identifying occult distant metastases which were not detected by conventional imaging. Only Corvera *et al *identified the role of ^18^F-FDG PET in detecting disease recurrences after resection in their analysis and reported that ^18^F-FDG PET was helpful to clarify recurrences with 76% of sensitivity [16]. Thus, ^18^F-FDG PET was merely confirmatory because recurrences were also identified on conventional imaging.

Unlike the aforementioned studies, the current analysis documented the complementary role of ^18^F-FDG PET/CT under a frequently encountered situation in the clinic in which clinicians suspected a recurrence. Of 50 patients, 36 had concordant results between ceCT and subsequent ^18^F-FDG PET/CT, and ^18^F-FDG PET/CT was 83% (30/36) consistent with the final recurrence status (Figure 2). Moreover, an additional PET/CT correctly identified six false-negative cases as recurrences which were interpreted as benign or equivocal by ceCT. This resulted in a significant increase in sensitivity (88% *vs*. 94%, *p *= 0.03) and suggested a complementary role of ^18^F-FDG PET/CT beyond ceCT when clinicians suspected a recurrence.

Of these six false-negative cases by ceCT, three were first interpreted as post-operative inflammatory changes. For the other three cases, lymph node metastasis was not confidently interpreted as a recurrence due to the insignificant size. However, the ^18^F-FDG PET/CT was interpreted as a recurrence in these lesions based on the high ^18^F-FDG uptake. From this information, five patients could receive palliative chemotherapy, whereas the other patient could not receive chemotherapy because of poor performance status. Moreover, an additional PET/CT showed no ^18^F-FDG uptake in five false-positive cases by ceCT, which provided decisive information to the clinicians. Among 14 patients with discordant results, 11 patients (11/14, 79%) was treated regarding to the results of PET/CT. In this context, when ceCT is inconclusive for identifying post-operative changes and the lymph nodes are of borderline size, a discernible role for ^18^F-FDG PET/CT is feasible.

This complementary role of ^18^F-FDG PET/CT during surveillance has been assessed in other cancers. Specifically, Radan *et al *described patients with breast cancer and rising tumor markers in which ^18^F-FDG PET/CT was superior to CT for diagnosis of tumor recurrence, and led to changes in the subsequent clinical management of 51% of the patients [19]. In addition, Guo *et al *evaluated the role of ^18^F-FDG PET/CT in patients with possibly recurrent esophageal squamous cell carcinoma who underwent definitive treatment and displayed a remarkable sensitivity and a high specificity and accuracy at regional and distant sites for recurrent esophageal squamous cell carcinoma [20].

Furukama *et al. *evaluated the prognostic significance of FDG uptake on PET in patients with biliary tract cancer and demonstrated the SUV_max _of 6.3 to be the optimal cutoff point for survival [21]. In addition, their data showed that the SUV_max _was one of the significant prognostic factors for overall survival in univariate analysis. However, our data did not show that the SUV_max _was the significant prognostic factor for overall survival, probably due to the low number of patients and selection bias.

Our study had limitations other than retrospective design. First of all, the majority of recurrent cases were confirmed clinically. Our standard for non-recurrence required no significant changes in the lesion for a minimum of 3 months; however, such a criterion has not been validated and may be insufficient for confirmation. Second, the validity of ^18^F-FDG PET/CT may have been overestimated because the nuclear medicine physicians were aware of the findings of the corresponding ceCT. Lastly, the longer time interval between ceCT and PET/CT and the different characteristics of 3 CT scanners between the period of 2003 to 2008 might influence the results. Nevertheless, our study has significance in demonstrating the complementary role of ^18^F-FDG PET/CT in a commonly encountered clinical situation in which clinicians suspect BTC recurrence by elusive clinical manifestations with equivocal or inconclusive conventional imaging. Additionally, as the most modern PET/CT scanners allow for fully diagnostic ceCT scans, contrast enhanced diagnostic PET/CT could be recommended as the primary modality of choice in case of suspected recurrence.

## Conclusions

We demonstrated that ^18^F-FDG PET/CT significantly improved the overall sensitivity of detecting BTC recurrences when ceCT was inconclusive and did not correspond to the clinical presentation. And ^18^F-FDG PET/CT was as sensitive and specific as ceCT in the detection of recurrent BTCs. Therefore, if both image modalities are discordant, ^18^F-FDG PET/CT is worthy of being weighted. Further prospective studies are necessary to establish the role of ^18^F-FDG PET/CT during surveillance.

## List of Abbreviations

The following abbreviations are used in this paper.

BTC: biliary tract cancer; ceCT: contrast-enhanced computed tomography; ^18^F-FDG PET/CT: ^18^F-fluorodeoxyglucose positron emission tomography integrated with computed tomography; SUV_max_: maximal standardized uptake value.

## Competing interests

The authors declare that they have no competing interests.

## Authors' contributions

YGL contributed to the study design, data collection/analysis/interpretation and writing of the manuscript. DY Oh treated many of the patients and contributed to the study design, data analysis/interpretation and writing of the manuscript. SWH, EKC, JYJ, SAITYK, SW K, SWH and YJB treated many of the patients in this study. All authors read and approved the final manuscript.

## Pre-publication history

The pre-publication history for this paper can be accessed here:

http://www.biomedcentral.com/1471-2407/11/188/prepub
